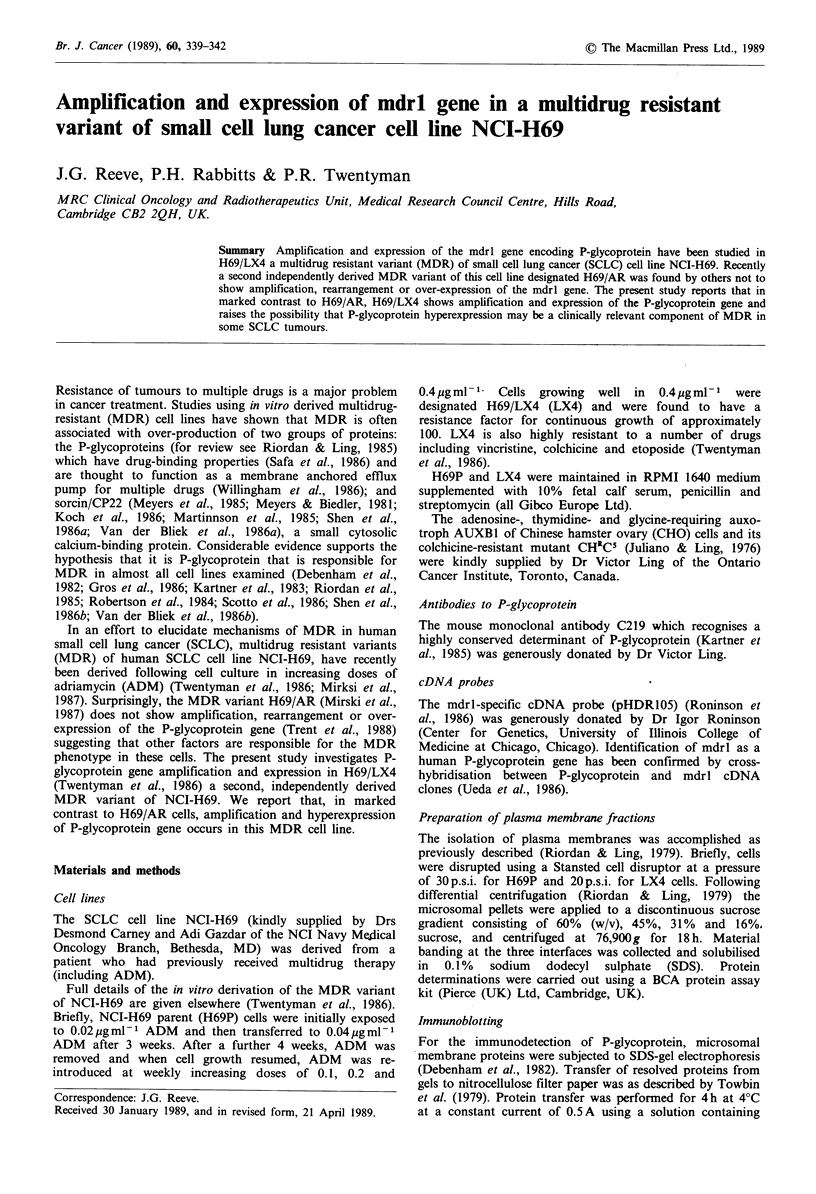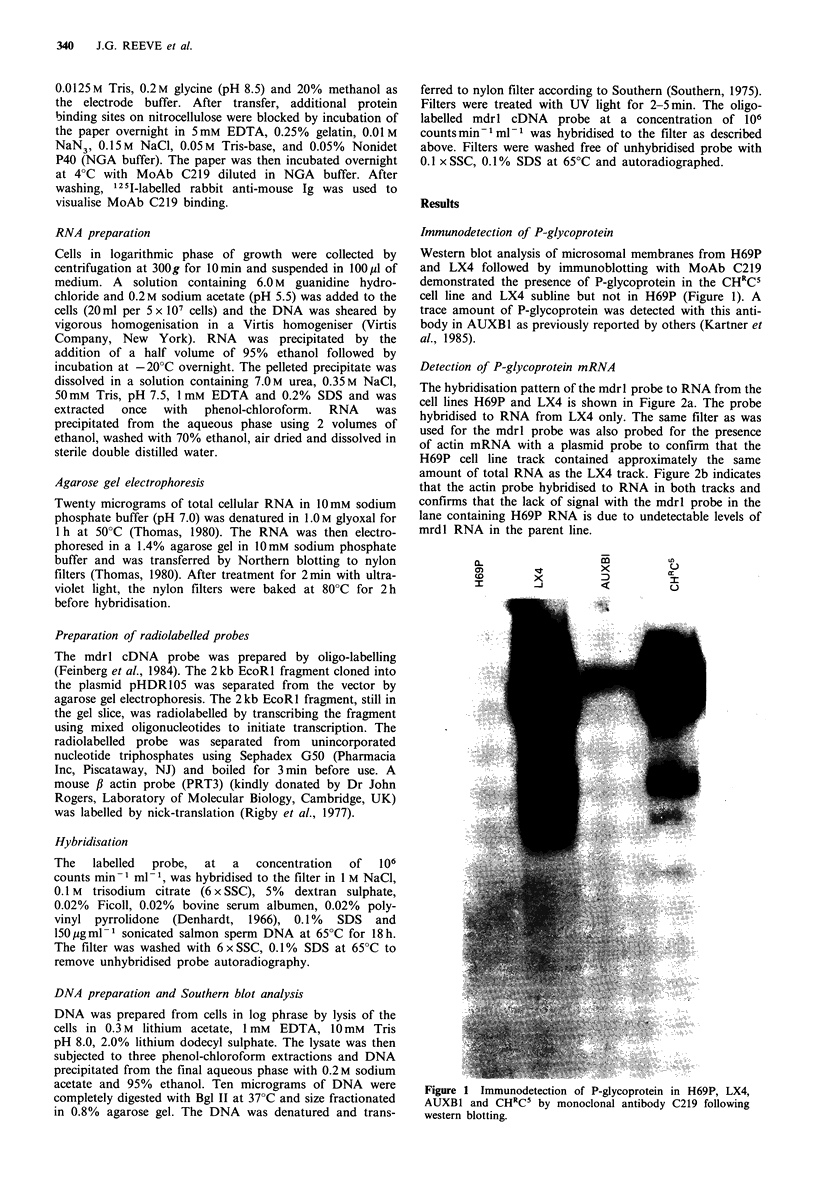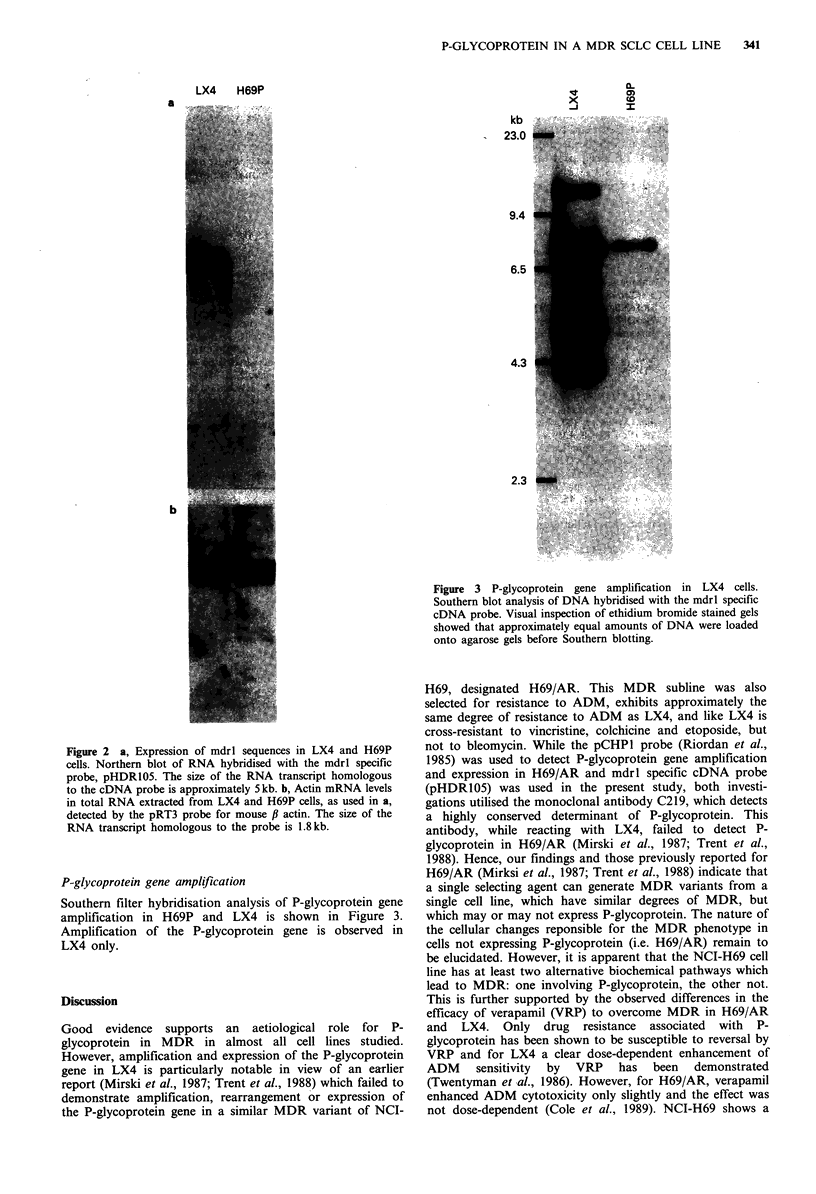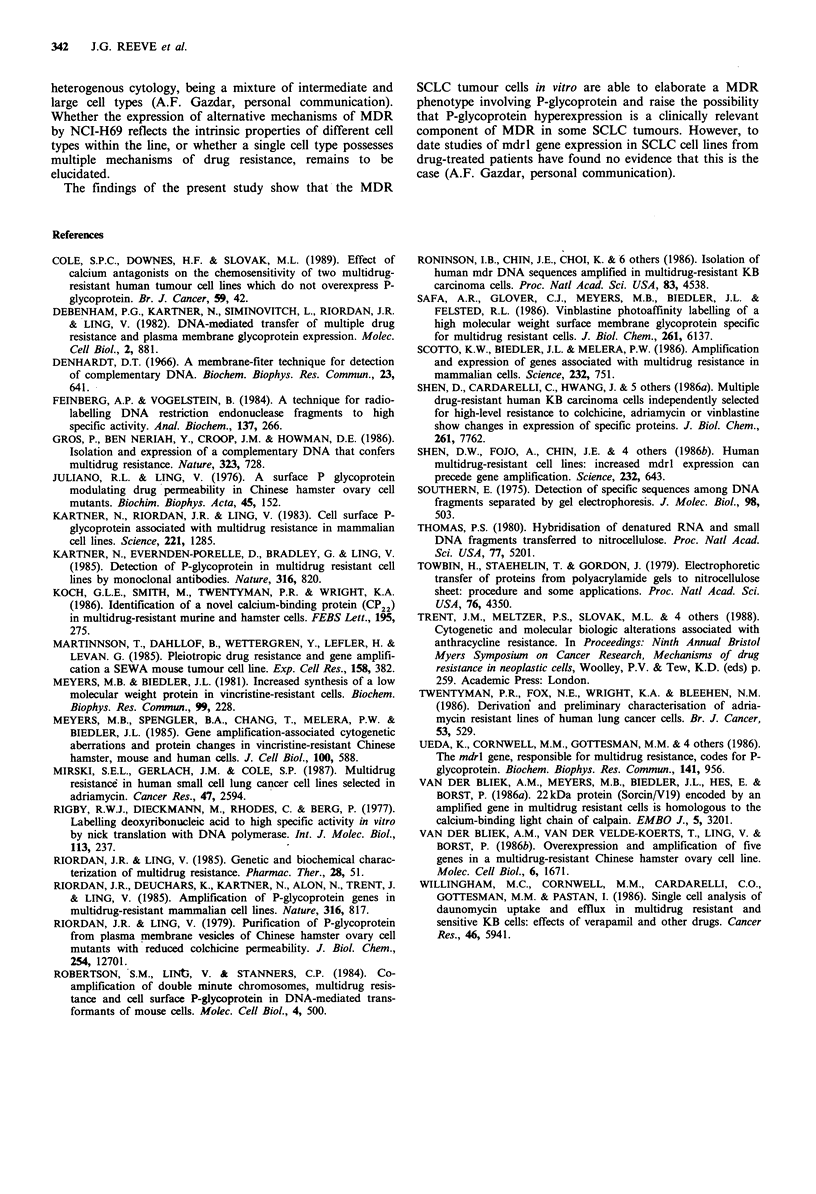# Amplification and expression of mdr1 gene in a multidrug resistant variant of small cell lung cancer cell line NCI-H69.

**DOI:** 10.1038/bjc.1989.282

**Published:** 1989-09

**Authors:** J. G. Reeve, P. H. Rabbitts, P. R. Twentyman

**Affiliations:** MRC Clinical Oncology and Radiotherapeutics Unit, Medical Research Council Centre, Cambridge, UK.

## Abstract

**Images:**


Br. J. Cancer (1989), 60, 339–342 © The Macmillan Press Ltd., 1989

### Amplification and expression of mdr1 gene in a multidrug resistant variant of small cell lung cancer cell line NCI-H69

J.G. Reeve, P.H. Rabbitts & P.R. Twentyman

*MRC Clinical Oncology and Radiotherapeutics Unit, Medical Research Council Centre, Hills Road, Cambridge CB2 2QH, UK.*

**Summary** Amplification and expression of the mdr1 gene encoding P-glycoprotein have been studied in H69/LX4 a multidrug resistant variant (MDR) of small cell lung cancer (SCLC) cell line NCI-H69. Recently a second independently derived MDR variant of this cell line designated H69/AR was found by others not to show amplification, rearrangement or over-expression of the mdr1 gene. The present study reports that in marked contrast to H69/AR, H69/LX4 shows amplification and expression of the P-glycoprotein gene and raises the possibility that P-glycoprotein hyperexpression may be a clinically relevant component of MDR in some SCLC tumours.

Resistance of tumours to multiple drugs is a major problem in cancer treatment. Studies using *in vitro* derived multidrug-resistant (MDR) cell lines have shown that MDR is often associated with over-production of two groups of proteins: the P-glycoproteins (for review see Riordan & Ling, 1985) which have drug-binding properties (Safa *et al*., 1986) and are thought to function as a membrane anchored efflux pump for multiple drugs (Willingham *et al*., 1986); and sorcin/CP22 (Meyers *et al*., 1985; Meyers & Biedler, 1981; Koch *et al*., 1986; Martinnson *et al*., 1985; Shen *et al*., 1986*a*; Van der Bliek *et al*., 1986*a*), a small cytosolic calcium-binding protein. Considerable evidence supports the hypothesis that it is P-glycoprotein that is responsible for MDR in almost all cell lines examined (Debenham *et al*., 1982; Gros *et al*., 1986; Kartner *et al*., 1983; Riordan *et al*., 1985; Robertson *et al*., 1984; Scotto *et al*., 1986; Shen *et al*., 1986*b*; Van der Bliek *et al*., 1986*b*).

In an effort to elucidate mechanisms of MDR in human small cell lung cancer (SCLC), multidrug resistant variants (MDR) of human SCLC cell line NCI-H69, have recently been derived following cell culture in increasing doses of adriamycin (ADM) (Twentyman *et al*., 1986; Mirksi *et al*., 1987). Surprisingly, the MDR variant H69/AR (Mirski *et al*., 1987) does not show amplification, rearrangement or over-expression of the P-glycoprotein gene (Trent *et al*., 1988) suggesting that other factors are responsible for the MDR phenotype in these cells. The present study investigates P-glycoprotein gene amplification and expression in H69/LX4 (Twentyman *et al*., 1986) a second, independently derived MDR variant of NCI-H69. We report that, in marked contrast to H69/AR cells, amplification and hyperexpression of P-glycoprotein gene occurs in this MDR cell line.

### Materials and methods

### Cell lines

The SCLC cell line NCI-H69 (kindly supplied by Drs Desmond Carney and Adi Gazdar of the NCI Navy Medical Oncology Branch, Bethesda, MD) was derived from a patient who had previously received multidrug therapy (including ADM).

Full details of the *in vitro* derivation of the MDR variant of NCI-H69 are given elsewhere (Twentyman *et al*., 1986). Briefly, NCI-H69 parent (H69P) cells were initially exposed to $0.02\,\mu g\,ml^{-1}$ ADM and then transferred to $0.04\,\mu g\,ml^{-1}$ ADM after 3 weeks. After a further 4 weeks, ADM was removed and when cell growth resumed, ADM was re-introduced at weekly increasing doses of 0.1, 0.2 and $0.4\,\mu g\,ml^{-1}$. Cells growing well in $0.4\,\mu g\,ml^{-1}$ were designated H69/LX4 (LX4) and were found to have a resistance factor for continuous growth of approximately 100. LX4 is also highly resistant to a number of drugs including vincristine, colchicine and etoposide (Twentyman *et al*., 1986).

H69P and LX4 were maintained in RPMI 1640 medium supplemented with 10% fetal calf serum, penicillin and streptomycin (all Gibco Europe Ltd).

The adenosine-, thymidine- and glycine-requiring auxotroph AUXB1 of Chinese hamster ovary (CHO) cells and its colchicine-resistant mutant $CH^RC^5$ (Juliano & Ling, 1976) were kindly supplied by Dr Victor Ling of the Ontario Cancer Institute, Toronto, Canada.

### Antibodies to P-glycoprotein

The mouse monoclonal antibody C219 which recognises a highly conserved determinant of P-glycoprotein (Kartner *et al*., 1985) was generously donated by Dr Victor Ling.

### cDNA probes

The mdr1-specific cDNA probe (pHDR105) (Roninson *et al*., 1986) was generously donated by Dr Igor Roninson (Center for Genetics, University of Illinois College of Medicine at Chicago, Chicago). Identification of mdr1 as a human P-glycoprotein gene has been confirmed by cross-hybridisation between P-glycoprotein and mdr1 cDNA clones (Ueda *et al*., 1986).

### Preparation of plasma membrane fractions

The isolation of plasma membranes was accomplished as previously described (Riordan & Ling, 1979). Briefly, cells were disrupted using a Stansted cell disruptor at a pressure of 30 p.s.i. for H69P and 20 p.s.i. for LX4 cells. Following differential centrifugation (Riordan & Ling, 1979) the microsomal pellets were applied to a discontinuous sucrose gradient consisting of 60% (w/v), 45%, 31% and 16% sucrose, and centrifuged at 76,900 *g* for 18 h. Material banding at the three interfaces was collected and solubilised in 0.1% sodium dodecyl sulphate (SDS). Protein determinations were carried out using a BCA protein assay kit (Pierce (UK) Ltd, Cambridge, UK).

### Immunoblotting

For the immunodetection of P-glycoprotein, microsomal membrane proteins were subjected to SDS-gel electrophoresis (Debenham *et al*., 1982). Transfer of resolved proteins from gels to nitrocellulose filter paper was as described by Towbin *et al*. (1979). Protein transfer was performed for 4 h at 4°C at a constant current of 0.5 A using a solution containing

Correspondence: J.G. Reeve.
Received 30 January 1989, and in revised form, 21 April 1989.

0.0125 M Tris, 0.2 M glycine (pH 8.5) and 20% methanol as the electrode buffer. After transfer, additional protein binding sites on nitrocellulose were blocked by incubation of the paper overnight in 5 mM EDTA, 0.25% gelatin, 0.01 M $NaN_3$, 0.15 M NaCl, 0.05 M Tris-base, and 0.05% Nonidet P40 (NGA buffer). The paper was then incubated overnight at 4°C with MoAb C219 diluted in NGA buffer. After washing, $^{125}$I-labelled rabbit anti-mouse Ig was used to visualise MoAb C219 binding.

*RNA preparation*

Cells in logarithmic phase of growth were collected by centrifugation at 300 *g* for 10 min and suspended in 100 $\mu$l of medium. A solution containing 6.0 M guanidine hydrochloride and 0.2 M sodium acetate (pH 5.5) was added to the cells (20 ml per $5\times10^7$ cells) and the DNA was sheared by vigorous homogenisation in a Virtis homogeniser (Virtis Company, New York). RNA was precipitated by the addition of a half volume of 95% ethanol followed by incubation at −20°C overnight. The pelleted precipitate was dissolved in a solution containing 7.0 M urea, 0.35 M NaCl, 50 mM Tris, pH 7.5, 1 mM EDTA and 0.2% SDS and was extracted once with phenol-chloroform. RNA was precipitated from the aqueous phase using 2 volumes of ethanol, washed with 70% ethanol, air dried and dissolved in sterile double distilled water.

*Agarose gel electrophoresis*

Twenty micrograms of total cellular RNA in 10 mM sodium phosphate buffer (pH 7.0) was denatured in 1.0 M glyoxal for 1 h at 50°C (Thomas, 1980). The RNA was then electrophoresed in a 1.4% agarose gel in 10 mM sodium phosphate buffer and was transferred by Northern blotting to nylon filters (Thomas, 1980). After treatment for 2 min with ultraviolet light, the nylon filters were baked at 80°C for 2 h before hybridisation.

*Preparation of radiolabelled probes*

The mdr1 cDNA probe was prepared by oligo-labelling (Feinberg *et al.*, 1984). The 2 kb EcoR1 fragment cloned into the plasmid pHDR105 was separated from the vector by agarose gel electrophoresis. The 2 kb EcoR1 fragment, still in the gel slice, was radiolabelled by transcribing the fragment using mixed oligonucleotides to initiate transcription. The radiolabelled probe was separated from unincorporated nucleotide triphosphates using Sephadex G50 (Pharmacia Inc, Piscataway, NJ) and boiled for 3 min before use. A mouse $\beta$ actin probe (PRT3) (kindly donated by Dr John Rogers, Laboratory of Molecular Biology, Cambridge, UK) was labelled by nick-translation (Rigby *et al.*, 1977).

*Hybridisation*

The labelled probe, at a concentration of $10^6$ counts $min^{-1}$ $ml^{-1}$, was hybridised to the filter in 1 M NaCl, 0.1 M trisodium citrate ($6\times$SSC), 5% dextran sulphate, 0.02% Ficoll, 0.02% bovine serum albumen, 0.02% polyvinyl pyrrolidone (Denhardt, 1966), 0.1% SDS and 150 $\mu g\,ml^{-1}$ sonicated salmon sperm DNA at 65°C for 18 h. The filter was washed with $6\times$SSC, 0.1% SDS at 65°C to remove unhybridised probe autoradiography.

*DNA preparation and Southern blot analysis*

DNA was prepared from cells in log phrase by lysis of the cells in 0.3 M lithium acetate, 1 mM EDTA, 10 mM Tris pH 8.0, 2.0% lithium dodecyl sulphate. The lysate was then subjected to three phenol-chloroform extractions and DNA precipitated from the final aqueous phase with 0.2 M sodium acetate and 95% ethanol. Ten micrograms of DNA were completely digested with Bgl II at 37°C and size fractionated in 0.8% agarose gel. The DNA was denatured and transferred to nylon filter according to Southern (Southern, 1975). Filters were treated with UV light for 2–5 min. The oligo-labelled mdr1 cDNA probe at a concentration of $10^6$ counts $min^{-1}$ $ml^{-1}$ was hybridised to the filter as described above. Filters were washed free of unhybridised probe with $0.1\times$SSC, 0.1% SDS at 65°C and autoradiographed.

### Results

*Immunodetection of P-glycoprotein*

Western blot analysis of microsomal membranes from H69P and LX4 followed by immunoblotting with MoAb C219 demonstrated the presence of P-glycoprotein in the $CH^RC^5$ cell line and LX4 subline but not in H69P (Figure 1). A trace amount of P-glycoprotein was detected with this antibody in AUXB1 as previously reported by others (Kartner *et al.*, 1985).

*Detection of P-glycoprotein mRNA*

The hybridisation pattern of the mdr1 probe to RNA from the cell lines H69P and LX4 is shown in Figure 2a. The probe hybridised to RNA from LX4 only. The same filter as was used for the mdr1 probe was also probed for the presence of actin mRNA with a plasmid probe to confirm that the H69P cell line track contained approximately the same amount of total RNA as the LX4 track. Figure 2b indicates that the actin probe hybridised to RNA in both tracks and confirms that the lack of signal with the mdr1 probe in the lane containing H69P RNA is due to undetectable levels of mrd1 RNA in the parent line.

**Figure 1** Immunodetection of P-glycoprotein in H69P, LX4, AUXB1 and $CH^RC^5$ by monoclonal antibody C219 following western blotting.

**Figure 2** **a**, Expression of mdr1 sequences in LX4 and H69P cells. Northern blot of RNA hybridised with the mdr1 specific probe, pHDR105. The size of the RNA transcript homologous to the cDNA probe is approximately 5 kb. **b**, Actin mRNA levels in total RNA extracted from LX4 and H69P cells, as used in **a**, detected by the pRT3 probe for mouse $\beta$ actin. The size of the RNA transcript homologous to the probe is 1.8 kb.

*P-glycoprotein gene amplification*

Southern filter hybridisation analysis of P-glycoprotein gene amplification in H69P and LX4 is shown in Figure 3. Amplification of the P-glycoprotein gene is observed in LX4 only.

### Discussion

Good evidence supports an aetiological role for P-glycoprotein in MDR in almost all cell lines studied. However, amplification and expression of the P-glycoprotein gene in LX4 is particularly notable in view of an earlier report (Mirski *et al.*, 1987; Trent *et al.*, 1988) which failed to demonstrate amplification, rearrangement or expression of the P-glycoprotein gene in a similar MDR variant of NCI-H69, designated H69/AR. This MDR subline was also selected for resistance to ADM, exhibits approximately the same degree of resistance to ADM as LX4, and like LX4 is cross-resistant to vincristine, colchicine and etoposide, but not to bleomycin. While the pCHP1 probe (Riordan *et al.*, 1985) was used to detect P-glycoprotein gene amplification and expression in H69/AR and mdr1 specific cDNA probe (pHDR105) was used in the present study, both investigations utilised the monoclonal antibody C219, which detects a highly conserved determinant of P-glycoprotein. This antibody, while reacting with LX4, failed to detect P-glycoprotein in H69/AR (Mirski *et al.*, 1987; Trent *et al.*, 1988). Hence, our findings and those previously reported for H69/AR (Mirksi *et al.*, 1987; Trent *et al.*, 1988) indicate that a single selecting agent can generate MDR variants from a single cell line, which have similar degrees of MDR, but which may or may not express P-glycoprotein. The nature of the cellular changes reponsible for the MDR phenotype in cells not expressing P-glycoprotein (i.e. H69/AR) remain to be elucidated. However, it is apparent that the NCI-H69 cell line has at least two alternative biochemical pathways which lead to MDR: one involving P-glycoprotein, the other not. This is further supported by the observed differences in the efficacy of verapamil (VRP) to overcome MDR in H69/AR and LX4. Only drug resistance associated with P-glycoprotein has been shown to be susceptible to reversal by VRP and for LX4 a clear dose-dependent enhancement of ADM sensitivity by VRP has been demonstrated (Twentyman *et al.*, 1986). However, for H69/AR, verapamil enhanced ADM cytotoxicity only slightly and the effect was not dose-dependent (Cole *et al.*, 1989). NCI-H69 shows a

**Figure 3** P-glycoprotein gene amplification in LX4 cells. Southern blot analysis of DNA hybridised with the mdr1 specific cDNA probe. Visual inspection of ethidium bromide stained gels showed that approximately equal amounts of DNA were loaded onto agarose gels before Southern blotting.

heterogenous cytology, being a mixture of intermediate and large cell types (A.F. Gazdar, personal communication). Whether the expression of alternative mechanisms of MDR by NCI-H69 reflects the intrinsic properties of different cell types within the line, or whether a single cell type possesses multiple mechanisms of drug resistance, remains to be elucidated.

The findings of the present study show that the MDR SCLC tumour cells *in vitro* are able to elaborate a MDR phenotype involving P-glycoprotein and raise the possibility that P-glycoprotein hyperexpression is a clinically relevant component of MDR in some SCLC tumours. However, to date studies of mdr1 gene expression in SCLC cell lines from drug-treated patients have found no evidence that this is the case (A.F. Gazdar, personal communication).

### References

COLE, S.P.C., DOWNES, H.F. & SLOVAK, M.L. (1989). Effect of calcium antagonists on the chemosensitivity of two multidrug-resistant human tumour cell lines which do not overexpress P-glycoprotein. *Br. J. Cancer*, **59**, 42.

DEBENHAM, P.G., KARTNER, N., SIMINOVITCH, L., RIORDAN, J.R. & LING, V. (1982). DNA-mediated transfer of multiple drug resistance and plasma membrane glycoprotein expression. *Molec. Cell Biol.*, **2**, 881.

DENHARDT, D.T. (1966). A membrane-fiter technique for detection of complementary DNA. *Biochem. Biophys. Res. Commun.*, **23**, 641.

FEINBERG, A.P. & VOGELSTEIN, B. (1984). A technique for radiolabelling DNA restriction endonuclease fragments to high specific activity. *Anal. Biochem.*, **137**, 266.

GROS, P., BEN NERIAH, Y., CROOP, J.M. & HOWMAN, D.E. (1986). Isolation and expression of a complementary DNA that confers multidrug resistance. *Nature*, **323**, 728.

JULIANO, R.L. & LING, V. (1976). A surface P glycoprotein modulating drug permeability in Chinese hamster ovary cell mutants. *Biochim. Biophys. Acta*, **45**, 152.

KARTNER, N., RIORDAN, J.R. & LING, V. (1983). Cell surface P-glycoprotein associated with multidrug resistance in mammalian cell lines. *Science*, **221**, 1285.

KARTNER, N., EVERNDEN-PORELLE, D., BRADLEY, G. & LING, V. (1985). Detection of P-glycoprotein in multidrug resistant cell lines by monoclonal antibodies. *Nature*, **316**, 820.

KOCH, G.L.E., SMITH, M., TWENTYMAN, P.R. & WRIGHT, K.A. (1986). Identification of a novel calcium-binding protein ($CP_{22}$) in multidrug-resistant murine and hamster cells. *FEBS Lett.*, **195**, 275.

MARTINNSON, T., DAHLLOF, B., WETTERGREN, Y., LEFLER, H. & LEVAN. G. (1985). Pleiotropic drug resistance and gene amplification a SEWA mouse tumour cell line. *Exp. Cell Res.*, **158**, 382.

MEYERS, M.B. & BIEDLER, J.L. (1981). Increased synthesis of a low molecular weight protein in vincristine-resistant cells. *Biochem. Biophys. Res. Commun.*, **99**, 228.

MEYERS, M.B., SPENGLER, B.A., CHANG, T., MELERA, P.W. & BIEDLER, J.L. (1985). Gene amplification-associated cytogenetic aberrations and protein changes in vincristine-resistant Chinese hamster, mouse and human cells. *J. Cell Biol.*, **100**, 588.

MIRSKI, S.E.L., GERLACH, J.M. & COLE, S.P. (1987). Multidrug resistance in human small cell lung cancer cell lines selected in adriamycin. *Cancer Res.*, **47**, 2594.

RIGBY, R.W.J., DIECKMANN, M., RHODES, C. & BERG, P. (1977). Labelling deoxyribonucleic acid to high specific activity *in vitro* by nick translation with DNA polymerase. *Int. J. Molec. Biol.*, **113**, 237.

RIORDAN, J.R. & LING, V. (1985). Genetic and biochemical characterization of multidrug resistance. *Pharmac. Ther.*, **28**, 51.

RIORDAN, J.R., DEUCHARS, K., KARTNER, N., ALON, N., TRENT, J. & LING, V. (1985). Amplification of P-glycoprotein genes in multidrug-resistant mammalian cell lines. *Nature*, **316**, 817.

RIORDAN, J.R. & LING, V. (1979). Purification of P-glycoprotein from plasma membrane vesicles of Chinese hamster ovary cell mutants with reduced colchicine permeability. *J. Biol. Chem.*, **254**, 12701.

ROBERTSON, S.M., LING, V. & STANNERS, C.P. (1984). Co-amplification of double minute chromosomes, multidrug resistance and cell surface P-glycoprotein in DNA-mediated transformants of mouse cells. *Molec. Cell Biol.*, **4**, 500.

RONINSON, I.B., CHIN, J.E., CHOI, K. & 6 others (1986). Isolation of human mdr DNA sequences amplified in multidrug-resistant KB carcinoma cells. *Proc. Natl Acad. Sci. USA*, **83**, 4538.

SAFA, A.R., GLOVER, C.J., MEYERS, M.B., BIEDLER, J.L. & FELSTED, R.L. (1986). Vinblastine photoaffinity labelling of a high molecular weight surface membrane glycoprotein specific for multidrug resistant cells. *J. Biol. Chem.*, **261**, 6137.

SCOTTO, K.W., BIEDLER, J.L. & MELERA, P.W. (1986). Amplification and expression of genes associated with multidrug resistance in mammalian cells. *Science*, **232**, 751.

SHEN, D., CARDARELLI, C., HWANG, J. & 5 others (1986*a*). Multiple drug-resistant human KB carcinoma cells independently selected for high-level resistance to colchicine, adriamycin or vinblastine show changes in expression of specific proteins. *J. Biol. Chem.*, **261**, 7762.

SHEN, D.W., FOJO, A., CHIN, J.E. & 4 others (1986*b*). Human multidrug-resistant cell lines: increased mdr1 expression can precede gene amplification. *Science*, **232**, 643.

SOUTHERN, E. (1975). Detection of specific sequences among DNA fragments separated by gel electrophoresis. *J. Molec. Biol.*, **98**, 503.

THOMAS, P.S. (1980). Hybridisation of denatured RNA and small DNA fragments transferred to nitrocellulose. *Proc. Natl Acad. Sci. USA*, **77**, 5201.

TOWBIN, H., STAEHELIN, T. & GORDON, J. (1979). Electrophoretic transfer of proteins from polyacrylamide gels to nitrocellulose sheet: procedure and some applications. *Proc. Natl Acad. Sci. USA*, **76**, 4350.

TRENT, J.M., MELTZER, P.S., SLOVAK, M.L. & 4 others (1988). Cytogenetic and molecular biologic alterations associated with anthracycline resistance. In *Proceedings: Ninth Annual Bristol Myers Symposium on Cancer Research, Mechanisms of drug resistance in neoplastic cells*, Woolley, P.V. & Tew, K.D. (eds) p. 259. Academic Press: London.

TWENTYMAN, P.R., FOX, N.E., WRIGHT, K.A. & BLEEHEN, N.M. (1986). Derivation and preliminary characterisation of adriamycin resistant lines of human lung cancer cells. *Br. J. Cancer*, **53**, 529.

UEDA, K., CORNWELL, M.M., GOTTESMAN, M.M. & 4 others (1986). The *mdr*1 gene, responsible for multidrug resistance, codes for P-glycoprotein. *Biochem. Biophys. Res. Commun.*, **141**, 956.

VAN DER BLIEK, A.M., MEYERS, M.B., BIEDLER, J.L., HES, E. & BORST, P. (1986*a*). 22 kDa protein (Sorcin/V19) encoded by an amplified gene in multidrug resistant cells is homologous to the calcium-binding light chain of calpain. *EMBO J.*, **5**, 3201.

VAN DER BLIEK, A.M., VAN DER VELDE-KOERTS, T., LING, V. & BORST, P. (1986*b*). Overexpression and amplification of five genes in a multidrug-resistant Chinese hamster ovary cell line. *Molec. Cell Biol.*, **6**, 1671.

WILLINGHAM, M.C., CORNWELL, M.M., CARDARELLI, C.O., GOTTESMAN, M.M. & PASTAN, I. (1986). Single cell analysis of daunomycin uptake and efflux in multidrug resistant and sensitive KB cells: effects of verapamil and other drugs. *Cancer Res.*, **46**, 5941.